# External birth defects in southern Vietnam: a population-based study at the grassroots level of health care in Binh Thuan province

**DOI:** 10.1186/1471-2431-13-67

**Published:** 2013-04-30

**Authors:** Truong Hoang, Dung The Nguyen, Phuong Van Ngoc Nguyen, Dong A Tran, Yves Gillerot, Raymond Reding, Annie Robert

**Affiliations:** 1 Pham Ngoc Thach University of Medecine, 86/2 Thanh Thai Street, Ho Chi Minh City, District 10, Vietnam; 2 Center for Human Genetics, Université catholique de Louvain, Brussels, Belgium; 3 Université catholique de Louvain, Institut de Recherche Expérimentale et Clinique, Pôle de chirurgie expérimentale et transplantation (CHEX), Brussels, Belgium; 4 Université catholique de Louvain, Institut de Recherche Expérimentale et Clinique, Pôle d’épidémiologie et biostatistique (EPID) and Public Health School, Brussels, Belgium

**Keywords:** Birth defect, External birth defect, Population-based study, Southern of Vietnam, Live births, ICD-10, Limbs defect, Orofacial clefts

## Abstract

**Background:**

There currently exists no data on birth defects from population-based studies in Vietnam. Our study's aim was to assess external birth defect (EBD) prevalence among live newborns in Binh Thuan Province in Vietnam with the help of health workers at all levels of the health system.

**Methods:**

A 2-month training session for 452 health professionals (HP) practicing delivery care in 127 Commune Health Stations (CHS) and in 12 provincial or district hospitals (DH) was setup in 2006. After a successful 6-month pilot study, a one-year registry of EBDs was established in 2008. All live newborns were screened for EBDs within 24 hours after birth in all DH obstetric departments and in all CHSs. Trained local HPs collected information by filling out a predesigned form and by photographing the affected newborn. EBDs were coded using the International Classification of Diseases system-10, Clinical Modification. The study was repeated in 2010.

**Results:**

Throughout 2010, out of a total of 13,954 newborns, 84 cases with one or more EBDs were reported, representing an overall prevalence rate of 60.2 per 10,000 live births. The most common groups of EBDs were limbs (27.2/10,000), orofacial clefts (20.1/10,000) and the central nervous system (7.9/10,000).

**Conclusions:**

This first population-based study in Vietnam, which required coordination efforts at the local level, provides baseline prevalences of external birth defects. Data on EBDs from this study in southern Vietnam may be useful for setting up a regional population-based registry of birth defects in Vietnam.

## Background

The toll of birth defects worldwide has been recognized as a severe public health problem. Birth defects, affecting 2-3% of all infants, are a major cause of perinatal mortality and childhood morbidity in both developed and developing countries [1]–[5].

Many studies have reported the prevalence of congenital anomalies in developed countries. Available data on this matter is very rare, however, in developing countries. Moreover, the few studies available are based on hospital births over a period of time rather than on a population [1,6]–[8].

At the present time, there are only two organizations in cooperation with the WHOs Human Genetics Programme in order to establish a registry of birth defects. These organizations are: the International Clearinghouse for Birth Defects Surveillance and Research (ICBDSR), which has 46 members representing 31 countries spread across the five continents [9], and the European Registration of Congenital Anomalies and Twins (EUROCAT), which has 43 members in 23 countries [10].

EUROCAT registries follow standardized guidelines and use multiple-source case ascertainment methods. They include all infants, including still births (from 20 weeks gestation), with anomalies diagnosed within the first year of life. Most major birth defect types are included. Termination of pregnancy for foetal anomaly (TOPFA) is also included. The criteria that must be met by registries participating in EUROCAT include a definition of the population, data collection and ascertainment, definition and coding of defects, calculation of prevalence rates, and confidentiality [8].

There is currently no data on birth defects available at the population level in Vietnam. The country is lacking an organization that is responsible for the registry or surveillance of birth defects. However, Vietnam has strong policies that seek to provide equitable healthcare for its people, as well as a good primary healthcare structure. EBD surveillance is therefore possible.

**Figure 1:**
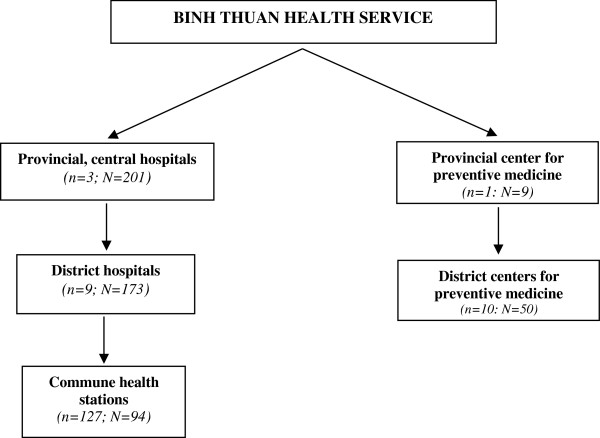
Structure of Binh Thuan’s health care system.

Binh Thuan is a rural province located along the south-eastern coast of Vietnam, with a population of approximately 1.1 million on an area of 7,992 km^2^, divided over 127 administrative communes. The population is over 90% Kinh ethnicity, which is the predominant ethnic group in Vietnam. As presented in Figure 1, Binh Thuan has an organizational health care structure at three levels: provincial or central health centers, district health centers and commune health stations (CHS).

All CHSs provide obstetric and mother-and -child healthcare, and implement prevention programs such as immunization and health promotion. However, there is less than one physician per CHS and most deliveries are supervised by midwives. Binh Thuan does not have a program for prenatal diagnosis at any level of healthcare. The good structure of healthcare at all levels, good access to primary care and a stable population representing the country's major ethnic group were all suitable conditions for implementing a population-based program for a birth defect registry within a population from the grassroots level to the topmost level of health care in Binh Thuan province.

Our study's aim was to assess EBD prevalence among live newborns in Binh Thuan Province in Vietnam with the help of health workers at all levels of the health system.

## Methods

A 2-month training session for 452 HP practicing delivery care in 127 Commune Health Stations (CHS) and in 12 provincial or district hospitals (DH) was setup in 2006. After a successful 6-months pilot study, a one-year registry of EBDs was established in 2008. After a few modifications in the setup, we conducted a whole registry in 2010.

In order to be included in this study, (1) the mother had to reside in the province, (2) the mother had to sign a consent form for enrolment in the study, (3) gestational age had to be at least 22 weeks, and (4) the baby had to be alive at birth.

All live births were physically examined to detect EBDs within 24 hours after birth in all DH obstetric departments and in all CHSs. When an EBD was suspected, a detailed clinical description of the EBD was collected and a photo of EBD was taken by a trained local health professional.

Trained local HPs collected information by filling out a predesigned form and by photographing the affected newborn. An external birth defects atlas and a manual for detecting EBDs in newborns were provided to each HP during training.

Data collection was performed by means of a structured form which contained three parts. The first part inquired on the mother's demographic profile, on medical and obstetric history, as well as on complications during the present pregnancy and labour. The second part pertained to neonatal characteristics, including sex, the Apgar score, gestational age, birth weight, head circumference, length, and history of birth defects in siblings. Finally, the third part was composed of a checklist for diagnosed congenital anomalies.

Birth-weight measurements were obtained at delivery using a scale (Testut, Paris, France) that was accurate to 10 grams. Infants were fully unclothed and in the supine position. Recumbent length was measured with a baby board (UNICEF) and recorded to the nearest 0.1cm. A non-flexible plastic tape was used for measuring head circumference of the newborns and the result was also recorded to the nearest 0.1 cm. The two latter measurements were obtained within 24 hours after delivery. The methods of measurements used were based on the recommendation of the WHO [11]. Absolute poverty certificates or poverty certificates, housing type, and personal income per month were used to classify maternal economic status.

EBDs were coded using the International Classification of Diseases system-10, Clinical Modification (ICD10-CM) [12] and common EBDs were pictured in an atlas along with a brief description of the defect.

Instructions for photographing and describing EBDs are contained in the manual. The manual also describes the technique for screening for EBDs in newborns during the physical exam.

All data (collection forms and photos of EBD cases) on live births were rechecked and entered into a Microsoft Excel sheet by a local trained data processor (a local health professional) before sending monthly data to a processing center at the Provincial Health Service.

All photographs with a written description of an EBD were reviewed and classified by one of the authors (Y.G) who is a Belgian expert in clinical genetics. In case of disagreement between the expert’s diagnosis and the local coding, the photograph and the written description were reviewed and discussed with local paediatric physicians. The team decided the most likely diagnosis as a group.

Statistical analyses were performed using the STATA statistical software.

Total prevalence was calculated by dividing the numerator (EBDs) by the relevant denominator (total live births) for the same period of time at the same place.

A newborn with multiple external defects was counted as one case unit for analysing birth defect case characteristics. When a newborn had more than one defect, each defect was counted as one unit when specific analyses for that particular defect or for system defect were performed. As a result, numerically adding up the number of defects could exceed the number of cases with defects.

The birth defect registry project was approved by the Binh Thuan Provincial Health Service and the Pham Ngoc Thach University Ethical Review Committee, Hochiminh City, Vietnam. All mothers provided written informed consent for themselves and for their baby prior to enrolment in the study, and consent to photograph was obtained from the parents of an affected newborn.

## Results

### Maternal socio-demographic characteristics

In 2010, a total of 13,954 newborns were registered, corresponding to a birth rate of 12.7 per one thousand people. The number of mothers was 13,877 because there were 71 pairs of twins (5.12 / 1,000 mothers) and 3 triple births (0.17 / 1,000 mothers). A caesarean section was performed for 17% of the deliveries.

**Figure 2:**
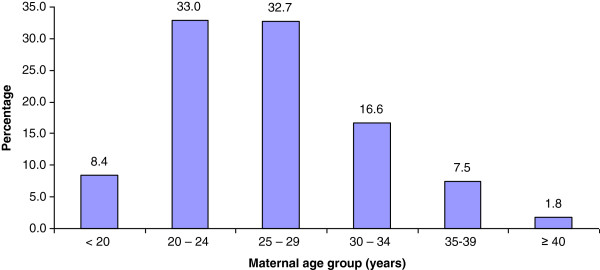
Histogram of maternal ages at delivery.

Mean maternal delivery age was 26.3 ± 5.5 years (mean ± SD) with a range of 13–50 years (Figure 2).

1.8% of mothers were aged over 40 years. 1,171 mothers (8.4%) were younger than 20 years, including 16 mothers who were 13 to 15 years.

Regarding maternal gravidity, gravidity 2–3 accounted for about half of mothers. Primigravida accounted for about a third (33.8%) of mothers and gravidity was 4 or more in 13.0%.

**Figure 3:**
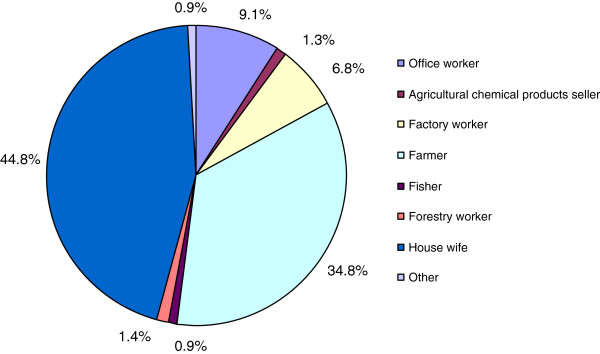
Distribution of mother’s occupations.

Figure 3 shows the distribution of professional activity of the mothers. Housewife and farmer accounted for over 80% of the mothers' occupations.

**Figure 4:**
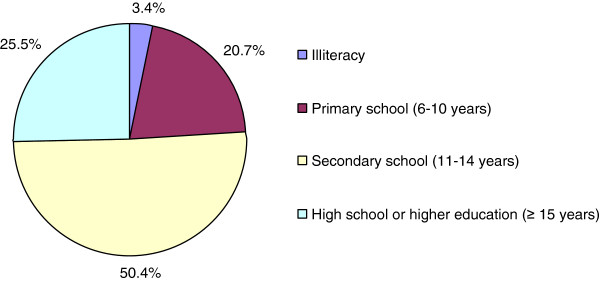
Distribution of maternal education level.

Half of the mothers had a secondary school or higher education degree, and 3.4% of mothers were illiterate (Figure 4).

There were about 2% of mothers living in absolute poverty, 7% of mothers living in poverty, and over 90% of mothers living in a better economic status.

Over 92% of mothers were from the Kinh ethnic group, and 70% of mothers lived in rural areas. Previous miscarriage was reported by 5% of mothers. Four babies were born from a mother in a consanguineous marriage (2.9/10,000 live births).

### Neonatal characteristics

There were 7,209 boys and 6,743 girls (sex ratio = 1.07). Two newborns had an indeterminate sex. The mean birth weight was 3,116 ± 432 g (mean ± SD) with a range of 400–5,300 g. Low birth weight accounted for 5% all live births. The mean gestational age for live births was 39.4 ± 1.6 weeks. About 8.2% of live births were premature. The mean birth length was 49.9 ± 2.3 cm (mean ± SD) with a range of 22–60 cm. The mean head circumference at birth was 32.6 ± 1.9 cm (mean ± SD) with a range of 16–60 cm for all live births. An Apgar score below 7 was observed in 6.4% of live births.

### External birth defect characteristics

There were 84 cases with one or more EBDs, representing an overall prevalence rate of 6.02 per 1,000 live births. In terms of sex distribution, 47.6% of the birth defect cases were boys (n=40), and 50.0% were girls (n=42).2.4% had ambiguous genitalia (n=2).

For mothers giving birth to a baby with external birth defects, the mean maternal delivery age was 27.0 ± 6.2 years (mean ± SD), with a range of 17–40 years.

**Figure 5:**
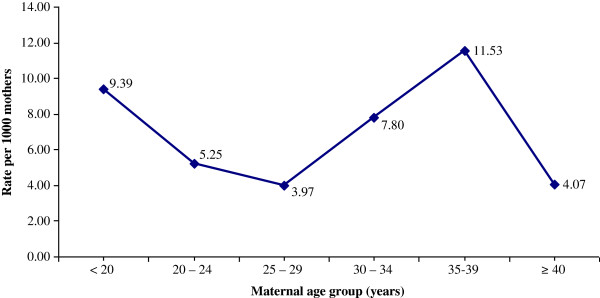
Distribution of the prevalence of external birth defect by maternal age at delivery.

The under 20 age group (9.39/1,000 mothers) and the 35–39 year age group (11.53/1,000) showed a 2.37 (95% CI: 1.09 - 4.95) and 2.91 (95% CI: 1.37 - 5.98) fold higher prevalence of overall external birth defects when compared to the 25–29 year age group (3.97/1,000), respectively (Figure 5).

External birth defects among women with primigravida and gravida 4 or more were 2.3 (95% CI: 1.43 - 3.70) and 2.2 (95% CI: 1.17 - 4.08) times higher than in women with gravida 2 or 3, respectively.

Regarding the maternal education level, the prevalence of EBDs was highest in the illiteracy group, and similar in other groups. Table 1 shows that the prevalence of EBDs decreased with increasing maternal economic status (trend test p-value = 0.03).

**Table 1:** Relationship between prevalence of external birth defects and selected maternal characteristics

**Maternal characteristics**	**External birth defects n = 84 (*%*)**	**Prevalence/1,000 mothers**	**PR^¥^**	**95% CI**
*Gravidity*				*p*= *0*.06*
1	41 (*44*.*8*)	8.75	2.31	1.43 – 3.75
2 -3	28 (*33*.*3*)	3.79	1	-
≥ 4	15 (*17*.*9*)	8.29	2.18	1.15 – 4.04
*Education level*				*p*= *0*.78*
Illiteracy	7 (*8*.*3*)	15.05	2.42	1.06 – 5.49
Primary school (6-10 years)	14 (*16*.*7*)	4.87	0.78	0.41 – 1.51
Secondary school (11–14 years)	41 (*48*.*8*)	5.86	0.94	0.57 – 1.60
High school or higher education (≥ 15 years)	22 (*26*.*2*)	6.22	1	-
*Economic status*				*p*= *0*.03*
Very poor	3 (*3*.*6*)	13.82	3.50	0.71 – 17.44
Poor	9 (*10*.*7*)	9.33	2.36	0.58 – 9.69
Well	70 (*83*.*3*)	5.74	1.46	0.43 – 5.39
Well-to-do	2 (*2*.*4*)	3.94	1	-
*Occupation of mother*				*p* = *0*.39*
Farmer and agricultural chemical product sellers	35 (*31*.*6*)	5,003	1.37	0.82 – 1.95
Other	49 (*63*.*9*)	8,874	1	-

* *Trend test p*-*value*; ^**¥**^ PR: *Prevalence ratio*.

The prevalence ratio (PR) of EBDs between literate mothers and illiterate mothers was 2.81 (95% CI: 1.02 - 5.76).

The EBD prevalence was higher in mothers with a history of miscarriage (9.40/1,000 live births vs. 6.37/1,000 live births for mothers without a previous miscarriage), in mothers who had a caesarean section (7.37/1,000 vs. 5.8/1,000 for mothers who delivered vaginally), in Kinh mothers (6.29/1,000 vs. 4.09/1,000 for other ethnicities), and in rural resident mothers (6.26/1,000 vs. 5.67/1,000 for urban resident mothers). These differences, however, were not significant between groups (Table 2).

**Table 2:** Relationship between prevalence of external birth defects and selected live births characteristics

**Live births characteristics**	**External birth defects n = 84 (%)**	**Prevalence 1,000 live births**	**PR**	**95% CI**	**P value**
*Birth weight*					
< 2500g	17 (*20*.*2*)	23.55	4.75	2.81 – 8.03	<0.001
≥ 2500g	67 (*79*.*8*)	5.06	1	-	
*Gestational age*					
< 37 weeks	13 (*15*.*5*)	11.61	2.09	1.17 – 3.27	0.012
≥ 37 weeks	71 (*84*.*5*)	5.57	1	-	
*AFGAR score at 5 minutes*					
< 7	21 (*25*.*0*)	23.60	4.89	2.95 – 7.87	<0.001
≥ 7	63 (*75*.*0*)	4.82	1	-	

The mean birth weight was 2,802 ± 593 g (mean ± SD) with a range of 1,000-4,300 g for babies presenting external birth defects. External birth defects were 4.75 times (95% CI: 2.81 - 8.03) more frequent among live births with low birth weight than among live births who weighed more than 2,500 g.

The mean gestational age for live births was 38.2 ± 3.5 weeks (mean ± SD) for babies with external birth defects. Babies born at less than 37 weeks of gestation were 2.09 times (95% CI: 1.17 - 3.27) more likely to have an external birth defect than babies born at 37 weeks or more.

EBDs were 4.89 times (95% CI: 2.95 – 7.87) more frequent among newborns with an Apgar score under 7 points at five minutes than among newborns with an Apgar score of 7 or more.

The mean birth length was 48.2 ± 4.2 cm (mean ± SD) with a range of 33–60 cm. The mean head circumference at birth was 31.4 ± 5.7 cm (mean ± SD) with a range of 16–60 cm for babies with external birth defects. There were no external birth defects in newborns from mothers in a consanguineous marriage (Table 3).

**Table 3:** Distribution of external birth defects across level of health care

**Level of health care**	**All live births**	**Overall external birth defects**	
	**N = 13, 954 (*%*)**	**n = 84 (*%*)**	**Per 1,000 live births**
Central hospital	9,026 (64.7)	58 (69.0)	6.43
District hospital	2,803 (20.1)	16 (19.1)	5.71
Commune health station	2,125 (15.2)	10 (11.9)	4.71

**Figure 6:**
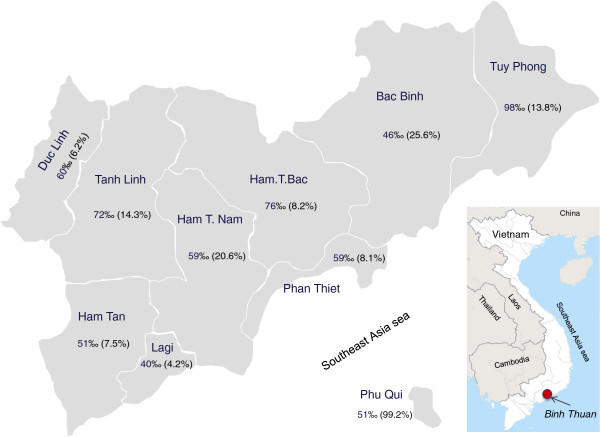
Birth defect distribution (‰) and proportion of babies born in commune health station (%) within each district.

Regarding level of health care, about one in seven (15.2%) infants was born outside of a hospital (Figure 6).

The most commonly encountered group of anomalies were limbs, which accounted for 27.2/10,000 live births, followed by orofacial clefts (20.1/10,000) and the central nervous system (7.9/10,000). Prevalence at birth for selected external birth defects is shown in Table 4 together with prevalences reported by full member EUROCAT registries, Belgium and Taiwan.

**Table 4:** **Prevalence of selected birth defects** (**per 10**,**000 live births**) **in Vietnam in comparison to the prevalences reported by full member EUROCAT registries**, **Belgium and Taiwan**

**Congenital anomalies subgroups**	**Vietnam**	**Full member EUROCAT**^**+**^	**Belgium**^**+**^	Taiwan^*^
*Nervous system*				
Neural Tube Defects				
Anencephalus and similar	3.58	3.50	3.16	1.07
Encephalocele	0.72	1.11	1.42	0.37
Spinabifida	0	4.74	4.89	0.58
Hydrocephaly	1.43	5.31	5.52	3.55
Microcephaly	2.15	2.37	1.74	0.58
Arhinencephaly/ holoprosencephaly	0	0.85	1.26	nr
*Ear*				
Anotia	0.72	0.34	0.47	nr
Microtia	2.15	nr	nr	nr
*Respiratory*				
Choanal atresia	0.72	0.79	0.95	0.21
*Orofacial clefts*				
Cleft lip with or without palate	14.33	8.63	12.00	12.80
Cleft palate	5.37	5.59	3.59	4.67
*Abdominal wall defects*				
Gastroschisis	1.43	2.82	1.89	1.20
*External genital system*				
Hypospadias	0.72	17.51	10.58	3.35
Indeterminate sex	1.43	0.59	0.63	0.99
*Limb*				
Limb reduction	4.30	5.05	5.05	3.22
Club foot	12.18	10.31	11.21	4.42
Polydactyly	6.45	8.69	6.79	7.97
Syndactyly	2.87	5.23	6.95	4.34
Other limb defects	1.43	nr	nr	nr

^+^ EUROCAT http://www.eurocat-network.eu/accessprevalencedata/prevalencetables. Date access 03/11/2013; * Data from reference 13; nr: none reported.

The prevalence of EBDs in Binh Thuan province was close to Taiwan data and not far from EUROCAT or Belgian data for most external birth defects. No cases of spinabifida were detected in our study.

## Discussion

The prevalence of birth defects can be influenced by many factors including case definition, TOPFA, the time of observation after birth, population study methods, case ascertainment methods and reporting and statistical procedures used [13]–[16].

Termination of pregnancy is legal up to 22 weeks in Vietnam, but reporting of pregnancy termination is not required. However, prenatal diagnosis does not exist in our study's population. We therefore believe that the reason for termination of pregnancy in Binh Thuan is rarely an external birth defect. Consequently, TOPFA most likely does not have an influence the prevalence of the birth defects.

We found that the prevalence of EBDs across the age distribution tended to be a U-shaped curve; prevalence dropped substantially for women over 40 years of age and only marginally for other age groups. For non-chromosomal defects, the U-shaped pattern of prevalence across maternal age has been documented by many authors [7,15,17].

In this study, the relationship between maternal education and an EBD did not necessarily mean that maternal education itself was a risk factor for EBDs. Educational qualification most probably determines socio-economic level and/or occupation and prenatal care behavior. It is therefore conceivable that education might affect the occurrence of EBDs indirectly [18].

Most reported associations between occupational exposures and adverse reproductive outcomes in epidemiological studies are equivocal and often controversial [19]. Significant association of occupational pesticide exposure and all birth defects were reported by Nurminen, et al. from a study in Finland [20], and by Restrepo et al. in Colombia [21]. Our findings show that the prevalence of EBDs was not significantly different between women involved in agricultural activities and/or working as an agricultural chemical products seller and mothers involved in another occupation.

Our results show an increased prevalence of external birth defects occurring among mothers with either primigravida or gravida over 4.

According to Swain et al., infants born to gravida 4 or more mothers have higher rate of birth defects when compared to mothers of lower gravidity [22]. Tan et al. reported that the prevalence of birth defect increased with birth order [23].

The relationship between the mother’s age at delivery and gravidity may be one possible explanation for the high rate of EBDs at both extremes of maternal gravidity in the present study.

This study demonstrates that birth defects are significantly associated with preterm birth and low birth weight. Although preterm and low birth weight infants are more likely to have birth defects, the effect of birth defects on preterm birth and low birth weight has been difficult to study because of multiple confounding risk factors [24,25].

Many studies have documented male preponderance in birth defects [26,27]. However, in the present study, a very slight female preponderance was found (42 females versus 40 males).

As expected, the overall prevalence of EBDs in our study (6.02 per 1000 live births) was lower than the EUROCAT (25.53/1000) and Belgian (23.11/1000) registries [11] because the present study reported only EBDs detected within 24 hours after birth. Our finding was similar to the prevalence rate in Taiwan, which is 7.3/1000 births. In Taiwan, EBDs were detected within a few days after birth [26].

When considering the type of external birth defect, limb defects, nervous system defects, orofacial clefts and external genital system defects are by far among the most common birth defects worldwide [28]–[31]. In the present study, limb defects, orofacial clefts and central nervous system defects were the three most common groups.

In our study, the most common limb defects were clubfoot, polydactyly and limb reduction, respectively.

Club foot is the common type of limb defect. Prevalence varies widely in among recent international reports. According to data from EUROCAT, the prevalence of clubfoot was reported to be 10.31/10,000 total births for all members, 11.21/10,000 in Belgium and varied from low (3.22 per 10,000) to high (18.00 per 10,000) in Ukraine and Saxony-Anhalt (Germany), respectively [10]. In recent studies in the United States, Parker et al. reviewed data from the 10 population-based birth defect surveillance programs (6,139 cases of clubfoot) to better estimate the prevalence of clubfoot and found the overall prevalence of clubfoot to be 19.2 per 10,000 live births [32]. Boo et al. reported an incidence of clubfoot in Malaysia at 45 per 10,000 live births [33]. In our study's group, club foot was the second most common EBD and the prevalence of 12.18/10,000 live births fell within the range reported for other registries.

Polydactyly is a major group. It is a defect that is easily detectable after birth and is an isolated finding in 85% - 88% of cases [34]. Our polydactyly prevalence of 6.45/10,000 live births was comparable to other European prevalence rates of 6.79/10,000 for Belgium, 6.80/ 10,000 for Paris, and 6.6/10,000 for Portugal respectively [10]. The prevalence of this birth defect is much higher in China (22.4/10, 000) [34] and in Alberta, Canada (18.84/10,000) [9]. Prevalence of polydactyly was reported to be lower in Barcelona, Spain (3.06/10,000) [10] and in Lombardy, Italy (5.82/10,000) [27].

Limb reduction is one of the most common types of limb defects and accounts for 3.2 to 7.06 per 10,000 births in the literature [10,26,27]. This very visible birth defect is symbolic because it launched the development of congenital anomalies surveillance activities worldwide after the thalidomide tragedy in the early 1960s. Limb reduction prevalence was found to be 4.3/10,000 live births among our newborns.

Orofacial clefts are among the most common of all major birth defects. Orofacial clefts are usually obviously visible immediately after birth.

Cleft lip with or without palate involved 20 out of 13,954 live births (14.33 per 10,000 live births), which is similar to the prevalence in Northern Ireland (14.70 per 10,000 live births) [10,27,35], lower than in Pakistan (19.10 per 10,000) [36] but higher than in full member EUROCAT registries (8.63 per 10, 000) [10], in Norway (10.9 per 10,000) [37], China (18.9 per 10,000) [38] and Korea (10.3 per 10,000) [39].

According the international perinatal database report on typical oral clefts, the prevalence of cleft lip with or without cleft palate from 54 registries in 30 countries over at least 1 complete year during the period 2000 to 2005 was 9.92 per 10,000 births, which was lower than our finding [40].

Isolated cleft palate is very difficult to detect prenatally due to shadowing artefacts from amniotic bands or other overlying structures.

The prevalence of 5.37 per 10,000 live births for cleft palate in this study was comparable to those observed in the full member EUROCAT registry (5.59 per 10,000) [10] and in Lombardy, Italy (5.82/10,000) [27]. Our figure was slightly higher than those reported in Taiwan (4.67 per 10, 000) [41], and in Belgium (3.59 per 10,000) [10], but lower than those reported in Wessex, United Kingdom (10.0/10 000) and in Ireland (7.21/10,000) [9].

Neural tube defects can be categorized as either anencephalus or similar (lack of closure in the head region) or spinabifida (lack of closure below the head). The two major categories of neural tube defects occur in approximately equal frequencies at birth [13,42].

Our data revealed that the prevalence for anencephalus or similar was 3.58 per 10,000 live births. This figure is comparable to that of the full member EUROCAT registry (3.50/10,000) and Belgium (3.16/10,000) [10]. In contrast with the relatively high frequencies of anencephaly, we did not observe any spinabifida in the present study or in the pilot study in 2008 with 16,593 births. The explanation for the absence of spinabifida cases in our study is complex. It may be due in part to the small sample size, the diagnostic technique used, and/or genetic factors.

As expected, the prevalence of hydrocephaly in our study (1.43/10,000 live births) was low compared to the full member EUROCAT registry (5.31/10,000), Belgium (5.52/10,000) and other registries [10,26,27,29,39]. Hydrocephaly is a malformation that is easier to diagnose by prenatal ultrasound scanning. It is not often obvious at birth and is usually detected after birth by an increasing head circumference that crosses percentiles on the growth chart. We therefore believe that hydrocephaly was under-diagnosed in our study.

Hypospadias is considered the most common congenital malformation in the genitourinary system. Usually hypospadias is detected at birth by a detailed examination of the newborn or by abnormal flow of urine during urination. Experienced clinical personnel are required to detect hypospadias. In our study, newborns were examined within 24 hour after birth by a local health provider with limited expertise in hypospadias recognition. Thus the prevalence of hypospadias was low (0.72/10,000 live births) compared to other registries [10,27,29,39].

The prevalence of external birth defects was not different between commune health stations and hospitals demonstrating health workers' abilities in detecting EBDs at commune health stations in Binh Thuan province.

Internal organ defects are not visible during a physical exam or they are often asymptomatic, particularly during the first 24 hours of life. In this study, since the examinations were executed by simple measurements and observations of the newborn, birth defects of internal organs (e.g. digestive system heart and circulatory system, internal urogenital system and certain domains of the central nervous system) were undetected.

## Conclusions

This first population-based study in Vietnam which required coordination efforts at the local level provides baseline prevalence of external birth defects. External birth defects can be diagnosed at birth; because our study was able to diagnose the majority of external birth defects occurring in Binh Thuan, the current data can be compared to the prevalence data of other registries.

Data on EBDs from this study in southern Vietnam may be useful in setting up a regional population-based registry of birth defects in Vietnam.

## Abbreviations

CHS: Commune health stations; DH: Provincial or District Hospitals; EBDs: External Birth Defects; EUROCAT: European Registration of Congenital Anomalies and Twins; HP: Health Professionals; ICD10-CM: International Classification of Diseases System-10, Clinical Modification; ICBDSR: International Clearinghouse for Birth Defects Surveillance and Research; TOPFA: Termination of Pregnancy For Foetal Anomaly.

## Competing interests

None of the authors of the above manuscripts has declared any conflict of interest statement.

## Authors’ contributions

TH participated in the design, carried out the study, performed the statistical analyses and drafted the manuscript. RR, TDA and YG provided advice in the design of the study and the analytical strategy and contributed to the manuscript revision. NNVP helped in the data analysis and report. AR and DNT are head of the project; they provided advice on the structure, the data analysis and presentation, and supervised the manuscript redaction. No author has any financial or private interest in this research project. There is no organization sponsoring this research which is granted by the Commission Universitaire pour le Développement (http://www.cud.be), which is a public funding from the Belgian government. The corresponding author has full access to all the data in the study and had final responsibility for the decision to submit for publication. All authors read and approved the final manuscript.

## Pre-publication history

The pre-publication history for this paper can be accessed here:

http://www.biomedcentral.com/1471-2431/13/67/prepub
